# Longitudinal relationships between visuomotor integration and physical fitness in preschoolers: a cross-lagged panel analysis

**DOI:** 10.3389/fpubh.2026.1804904

**Published:** 2026-06-18

**Authors:** Yu Liu, Xiaoyue Zhang, Zhicheng Lin, Zhixiu Lan, Linyan Dong, Baifa Zhang

**Affiliations:** 1 School of Sports, Southwest University, Chongqing, China; 2 Experimental Kindergarten of Southwest University, Chongqing, China

**Keywords:** children, cross-lagged panel analysis, physical fitness, preschooler, visuomotor integration

## Abstract

**Purpose:**

Physical fitness and visuomotor integration are developmentally significant constructs in early childhood. Although physical fitness and visuomotor integration are positively associated in preschool children, the majority of existing evidence is cross-sectional, precluding inferences about causal directionality. The present study employed a cross-lagged panel design to examine the longitudinal bidirectional relationships between physical fitness and visuomotor integration across a one-year period among preschoolers.

**Methods:**

A total of 202 preschool children (mean age = 4.65 ± 0.84 years) from a kindergarten in Chongqing, China, participated in a 12 month longitudinal study with assessments at baseline (T1) and follow-up (T2), of whom 194 completed the study (retention rate: 96.1%). Visuomotor integration was measured using the Beery VMI-6 short form, and physical fitness was assessed using a five-item test battery derived from China's National Physical Fitness Measurement Standards. Cross-lagged panel analysis was conducted to examine bidirectional associations between physical fitness and visuomotor integration, controlling for age and sex.

**Results:**

The cross-lagged model demonstrated adequate fit (χ^2^/df = 2.23, CFI = 0.972, TLI = 0.952, RMSEA = 0.073). Both constructs exhibited significant autoregressive stability (physical fitness: β = 0.88, visuomotor integration: β = 0.61, all *p* < 0.01). After controlling for age, sex, and autoregressive effects, T1 physical fitness significantly predicted T2 visuomotor integration (β = 0.27, *p* < 0.01), whereas the reverse path was not significant (β = 0.09, *p* = 0.13). Among the physical fitness indicators, standing long jump exhibited the highest factor loading at both time points, suggesting it may serve as a primary contributor to this predictive relationship.

**Conclusions:**

Physical fitness was longitudinally associated with subsequent visuomotor integration in preschool children. These findings contribute longitudinal evidence on the link between physical fitness and later visuomotor integration during early childhood.

## 1 Introduction

Early childhood professionals and curricula have long emphasized the importance of visuomotor integration (1, 2). For example, the Early Learning and Development Guidelines for Children Aged 3–6 (China) identifies it as a key dimension of fine motor development. Visuomotor integration (VMI) refers to the capacity to coordinate visuospatial perception and attentional control with fine motor output, enabling precise manual tasks such as drawing, writing, and copying geometric forms (3). Many foundational skills essential for academic performance, including comprehending instructions, sustaining attention, and manipulating writing utensils, are directly influenced by visuomotor integration (4). Children with well-developed visuomotor integration skills are better equipped to integrate visual representations with emerging literacy and numeracy skills through activities such as letter and number writing and object classification based on mathematical concepts (5). Early childhood, particularly ages 4 to 7, represents a phase of rapid visuomotor integration development, with growth peaking between ages 4 and 5 (6, 7), and fostering this capacity during this critical window lays the foundation for school readiness and subsequent academic success (4, 8).

A growing body of evidence suggests a positive association between physical fitness and visuomotor integration (9–12). Physical fitness is defined as an individual's capacity to perform physical activities, encompassing cardiorespiratory fitness, musculoskeletal fitness (power, strength, endurance, and flexibility), and motor fitness (skill-related attributes such as speed, agility, balance, and coordination) (13, 14). Given its developmental significance, children's physical fitness has been regularly monitored in China since 2000, with the National Physical Fitness Measurement (NPFM) assessing children aged 3–6 years (15). In a cross-sectional study of children aged 3–6 years, Liu et al. demonstrated that 20-m sprint performance, throwing performance, and visuomotor integration were significantly intercorrelated (10). Mediation analysis revealed that the 20 m sprint and standing long jump partially mediated the relationship between age and visuomotor integration. Furthermore, physical fitness and fundamental motor skills have been shown to be bidirectionally predictive in preschoolers (16), and gross motor exercises may enhance visuomotor integration during this developmental period (17, 18), whereas evidence on whether visuomotor integration reciprocally predicts physical fitness remains limited. However, the predominance of cross-sectional designs in current research precludes examining reciprocal longitudinal associations between physical fitness and visuomotor integration. Longitudinal studies are warranted to elucidate temporal relationships between these variables, thereby informing interventions targeting visuomotor integration in pediatric populations. Therefore, this study aimed to examine bidirectional relationships between physical fitness and visuomotor integration in preschool children using cross-lagged panel analysis. Based on prior evidence, we hypothesized that physical fitness would be positively associated with subsequent visuomotor integration.

## 2 Material and methods

### 2.1 Participants

The sample consisted of 202 preschool children (mean age = 4.65 ± 0.84 years) recruited through convenience sampling from a single kindergarten in Chongqing, China. The present study employed a two-wave, 12-month longitudinal design, with baseline assessment (T1) conducted in September 2024 and follow-up assessment (T2) in September 2025. Of the original sample, 194 children completed T2, yielding an attrition rate of 3.9% due to absences, illness, and school transfers. Written informed consent was obtained from the parents of all participants prior to data collection.

### 2.2 Instruments

#### 2.2.1 Visuomotor integration

Visuomotor integration was assessed using the Beery Visual-Motor Integration 6th Edition (Beery VMI), a standardized test first published in 1967 that requires children to copy geometric figures of progressive difficulty (19). The 21-item short form, designed for children aged 2 to 7 years, is administered using pencil and paper and requires approximately 10 min to complete. Administration is discontinued after three consecutive failures. Each correctly reproduced figure receives 1 point, yielding raw scores ranging from 0 to 21. The test was administered by postgraduate students majoring in physical education who had completed 1 week of training in administration procedures.

#### 2.2.2 Physical fitness

Physical fitness in preschool children was assessed using a comprehensive test battery derived from the Manual of the National Physical Fitness Measurement Standards (Early Childhood Section), issued by China's General Administration of Sport (2023). Five commonly used test items were selected from the manual for the current study, including the 15-m obstacle run, standing long jump, double-legged continuous jump, balance beam walk, and grip strength (20). The sit-and-reach test was excluded because it assesses flexibility, whereas the present study focused on fitness components more closely aligned with dynamic gross motor performance, namely muscular strength and power, balance, coordination, agility, and speed. All tests were administered twice, and the better score was recorded for analysis. Within-session test–retest reliability for the five physical fitness items was assessed using intraclass correlation coefficients computed from the two trials administered by the same examiner, with ICCs ranging from 0.88 to 0.90.

#### 2.2.3 Procedure

After obtaining informed consent, five trained research assistants administered individual visuomotor integration assessments to children in groups of five in a quiet classroom, with each session lasting approximately 10 min. Physical fitness assessments were conducted on the preschool playground. Trained research assistants were responsible for administering the tests, ensuring children's safety, and recording results. Before test administration, children were instructed to remain calm, avoid vigorous physical activity, and wear their preschool sports uniform. The test battery was arranged in order of increasing physical demand to minimize fatigue effects on subsequent measures: grip strength, balance beam walk, standing long jump, double-legged continuous jump, and 15-m obstacle run. Follow-up assessments were conducted by the same trained research team, using the same testing protocols, equipment, and testing environment as at baseline. Although assessors were not formally blinded to assessment wave, standardized administration and scoring procedures were applied at both waves to minimize measurement bias.

#### 2.2.4 Data processing and analysis

All analyses were performed using IBM SPSS Statistics version 23.0 (IBM Corp., Armonk, NY, USA) and R version 4.5.1 (R Foundation for Statistical Computing, Vienna, Austria). Descriptive statistics were computed for all study variables. Raw scores for the timed physical fitness tasks are presented in the descriptive tables, where lower scores indicate better performance. For the cross-lagged panel analysis, these variables were reverse-scored by multiplication by −1 so that higher values consistently indicated better performance across all physical fitness indicators. Prior to the primary analyses, preliminary analyses included paired-samples *t*-tests to compare variables between the two time points, independent-samples *t*-tests to examine sex differences, and Pearson correlation analyses to assess associations between physical fitness and visuomotor integration at both time points. Cross-lagged analysis was conducted to examine the temporal stability of physical fitness and visuomotor integration and their bidirectional longitudinal associations from baseline (T1) to follow-up (T2). Sex and age at T1 were included as time-invariant covariates predicting physical fitness and visuomotor integration at both time points. Residual covariances were specified between each physical fitness indicator and its T2 counterpart to account for indicator-specific variance over time. The robust maximum likelihood estimator (MLR) was employed to estimate model parameters. Missing data were handled with full information maximum likelihood under the assumption that data were missing at random, with all available data contributing to model estimation. Model goodness-of-fit was assessed using the chi-square to degrees of freedom ratio (χ^2^/df < 3), Comparative Fit Index (CFI > 0.90), Tucker–Lewis index (TLI > 0.90), and Root Mean Square Error of Approximation (RMSEA < 0.08), with values meeting these thresholds indicating acceptable fit (21). Statistical significance was set at α = 0.05 for all analyses, and all reported coefficients are standardized.

## 3 Results

### 3.1 Descriptive data and correlations

Descriptive statistics for all variables and independent samples *t*-test results for gender differences at both time points are presented in Table 1. Significant gender differences were observed at both time points (T1 and T2) for VMI and grip strength, with girls demonstrating higher VMI scores (T1: *p* = 0.02; T2: *p* < 0.01) and boys demonstrating greater grip strength (T1: *p* = 0.01; T2: *p* = 0.04).

**Table 1:** Independent samples *t*-test results by sex at T1 and T2.

Variable	Boys (*n* = 94)	Girls (*n* = 100)	*p*
Age (year; T1)	4.61 ± 0.83	4.71 ± 0.86	0.39
Grip strength (kg; T1)	4.69 ± 2.12	4.02 ± 1.86	0.02^*^
Grip strength (kg; T2)	6.57 ± 2.46	5.52 ± 2.19	< 0.01^**^
Balance beam walk (s; T1)	17.50 ± 14.85	18.24 ± 13.10	0.71
Balance beam walk (s; T2)	9.93 ± 8.97	12.21 ± 10.33	0.10
Standing long jump (cm; T1)	77.40 ± 22.00	76.00 ± 20.27	0.64
Standing long jump (cm; T2)	99.34 ± 21.79	93.72 ± 20.70	0.07
Double-legged continuous jump (s; T1)	7.82 ± 4.22	7.39 ± 3.45	0.43
Double-legged continuous jump (s; T2)	5.44 ± 1.19	5.48 ± 1.53	0.86
15-meter obstacle run (s; T1)	8.75 ± 1.82	9.09 ± 1.80	0.19
15-meter obstacle run (s; T2)	7.63 ± 0.99	7.72 ± 0.87	0.51
VMI (T1)	8.90 ± 3.54	10.20 ± 3.66	0.01^*^
VMI (T2)	10.56 ± 3.73	11.65 ± 3.66	0.04^*^

For timed tasks, lower scores indicate better performance; VMI, visuomotor integration; ^*^p < 0.05, ^**^p < 0.01.

In addition to the gender difference analysis, paired-samples *t*-tests were conducted to evaluate changes from T1 to T2 (Table 2). At the 12-month follow-up, all variables showed statistically significant increases.

**Table 2:** Changes between T1 and T2 data.

Variable	T1	T2	Variation	95% CI	*p*
Grip strength (kg)	4.35 ± 2.01	6.03 ± 2.38	1.68 ± 1.75	(1.43, 1.93)	< 0.01^**^
Balance beam walk (s)	17.88 ± 13.94	11.10 ± 9.74	6.78 ± 12.22	(5.05, 8.51)	< 0.01^**^
Standing long jump (cm)	76.68 ± 21.08	96.44 ± 21.37	19.76 ± 13.70	(17.82, 21.70)	< 0.01^**^
Double-legged continuous jump (s)	7.60 ± 3.84	5.46 ± 1.37	2.14 ± 3.46	(1.65, 2.63)	< 0.01^**^
15-m obstacle run (s)	8.92 ± 1.81	7.67 ± 0.93	1.25 ± 1.46	(1.04, 1.46)	< 0.01^**^
VMI	9.57 ± 3.65	11.12 ± 3.73	1.55 ± 2.23	(1.24, 1.87)	< 0.01^**^

For timed tasks, lower scores indicate better performance. VMI, visuomotor integration; ^*^p < 0.05, ^**^p < 0.01.

Table 3 presents the correlations among all study variables. All measures at T1 were significantly correlated with their corresponding measures at T2 (*r* = 0.44–0.82, *p* < 0.01), indicating moderate-to-high stability over time. Visuomotor integration was moderately associated with physical fitness both concurrently (*r* = 0.32–0.67, *p* < 0.01) and longitudinally (*r* = 0.30–0.63, *p* < 0.01).

**Table 3:** Correlation analysis at T1 and T2.

Variable	1	2	3	4	5	6	7	8	9	10	11	12
1. Grip strength (T1)	1											
2. Grip strength (T2)	0.69^**^	1										
3. Balance beam walk (T1)	0.36^**^	0.31^**^	1									
4. Balance beam walk (T2)	0.39^**^	0.33^**^	0.52^**^	1								
5. Standing long jump (T1)	0.64^**^	0.66^**^	0.46^**^	0.53^**^	1							
6. Standing long jump (T2)	0.56^**^	0.64^**^	0.43^**^	0.49^**^	0.79^**^	1						
7. Double-legged continuous jump (T1)	0.42^**^	0.50^**^	0.40^**^	0.47^**^	0.63^**^	0.54^**^	1					
8. Double-legged continuous jump (T2)	0.33^**^	0.33^**^	0.32^**^	0.40^**^	0.44^**^	0.43^**^	0.44^**^	1				
9.15-m obstacle run (T1)	0.55^**^	0.58^**^	0.37^**^	0.47^**^	0.69^**^	0.66^**^	0.69^**^	0.38^**^	1			
10.15-m obstacle run (T2)	0.45^**^	0.51^**^	0.45^**^	0.39^**^	0.57^**^	0.64^**^	0.57^**^	0.36^**^	0.60^**^	1		
11. VMI (T1)	0.50^**^	0.55^**^	0.35^**^	0.44^**^	0.67^**^	0.58^**^	0.57^**^	0.40^**^	0.56^**^	0.46^**^	1	
12. VMI (T2)	0.48^**^	0.54^**^	0.30^**^	0.36^**^	0.63^**^	0.50^**^	0.53^**^	0.39^**^	0.55^**^	0.32^**^	0.82^**^	1

Timed variables were reverse-scored (multiplied by −1) such that higher values indicate better performance; VMI, visuomotor integration; ^*^p < 0.05, ^**^p < 0.01.

### 3.2 Cross-lagged model results

**Figure 1:**
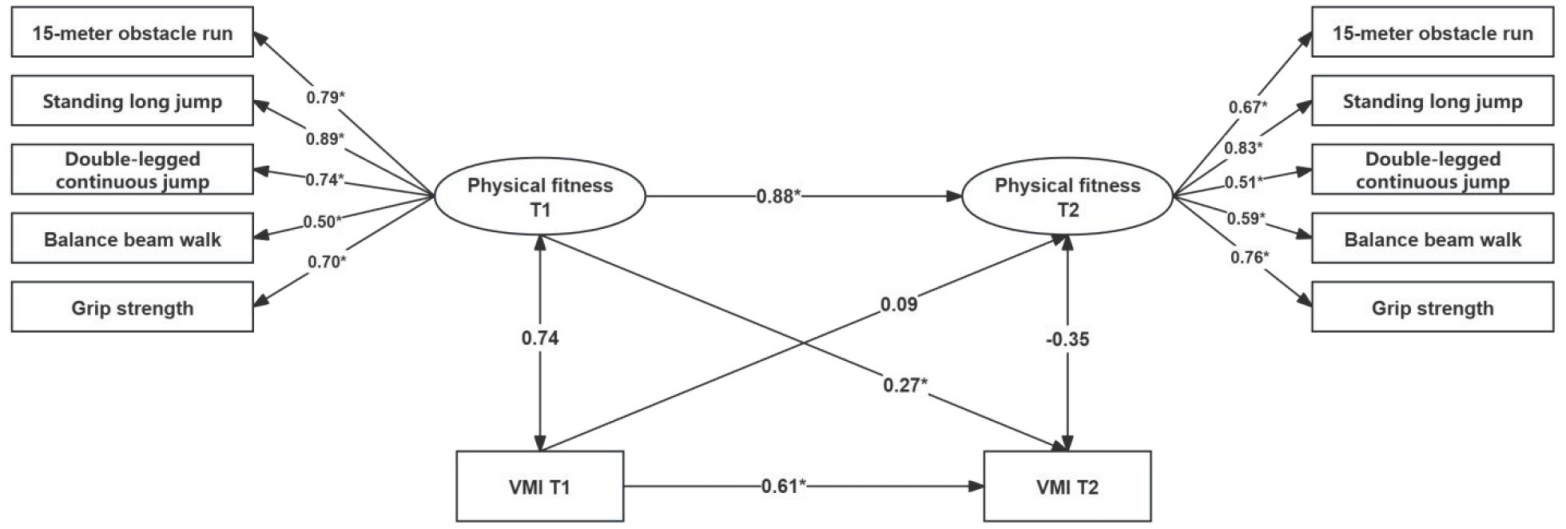
Cross-lagged model results for physical fitness and visuomotor integration. ^*^p < 0.05.

Longitudinal metric invariance of the physical fitness construct was tested by constraining the corresponding factor loadings to equality across T1 and T2. Metric invariance was supported, as the decrement in model fit relative to the configural model was negligible (ΔCFI = 0.005). The model demonstrated acceptable-to-good fit to the data: χ^2^ = 136.27, df = 61, χ^2^/df = 2.23, RMSEA = 0.073, CFI = 0.972, and TLI = 0.952. As illustrated in Figure 1, T1 physical fitness significantly predicted T2 physical fitness (β = 0.88, SE = 0.06, *p* < 0.01), and T1 visuomotor integration significantly predicted T2 visuomotor integration (β = 0.61, SE = 0.07, *p* < 0.01). Based on the β coefficients, physical fitness demonstrated greater temporal stability than visuomotor integration. After controlling for age, sex, and autoregressive effects, T1 physical fitness significantly predicted T2 visuomotor integration (β = 0.27, SE = 0.08, *p* < 0.01), whereas T1 visuomotor integration did not significantly predict T2 physical fitness (β = 0.09, SE = 0.06, *p* = 0.13). Among the physical fitness indicators, standing long jump exhibited the highest factor loading at both time points (T1: β = 0.89; T2: β = 0.83).

## 4 Discussion

This longitudinal study examined the bidirectional associations between visuomotor integration and physical fitness among preschool children over a 1-year period. The results revealed significant autoregressive paths for both visuomotor integration and physical fitness, indicating stability over time. Furthermore, the findings indicated that physical fitness, specifically standing long jump performance, significantly predicted subsequent visuomotor integration. However, no evidence of a bidirectional relationship was observed, as visuomotor integration did not significantly predict subsequent physical fitness.

The present study indicated that both physical fitness and visuomotor integration improved over the 12-month follow-up period, consistent with previous studies on developmental patterns in preschool children (10, 16, 22). Examination of gender differences revealed that boys exhibited greater grip strength, whereas girls demonstrated higher visuomotor integration scores. This gender difference in grip strength is consistent with a meta-analysis of 169 studies reporting that boys display greater grip strength than girls from birth to age 16, with girls aged 3–10 years demonstrating approximately 90% of boys' grip strength (23). However, the literature on gender differences in visuomotor integration remains inconsistent. For example, Fang et al. found that girls aged 4–6 years demonstrated significantly higher visuomotor integration performance than boys (7). In contrast, Liu et al. found no significant gender differences in visuomotor integration among preschool children aged 3–6 years (10), with some researchers suggesting that such differences may not emerge until third grade (24).

The cross-lagged panel analysis revealed that T1 physical fitness significantly predicted T2 visuomotor integration, whereas T1 visuomotor integration did not significantly predict T2 physical fitness, suggesting a unidirectional rather than bidirectional relationship between these constructs. This finding is consistent with Liu et al. (33), who reported that the 20 m fast run and standing long jump partially mediated the relationship between age and visuomotor integration in preschool children aged 3–6 years. The present study provides longitudinal evidence that physical fitness is associated with subsequent visuomotor integration in preschool children. One possible explanation involves the shared neural substrates underlying motor and cognitive functions. From a neuropsychological perspective, the cerebellum, prefrontal cortex, basal ganglia, and associated structures exhibit strong functional coupling, as these regions are co-activated during both motor and cognitive tasks (25, 26). Visuomotor integration encompasses multiple cognitive and neuromotor processes, including visuospatial perception, visual size discrimination, visual retrieval, and orientation discrimination (27). According to Piaget's theoretical framework concerning the parallel development of motor and cognitive domains (28), physically fitter children may engage in greater physical activity than their less fit peers (29), thereby expanding opportunities for environmental exploration and motor skill acquisition, which may subsequently enhance the development of visuomotor integration related cognitive processes (11). Consistent with this view, Africa et al. (17) found that a 14-week gross motor activity program yielded greater improvements in visuomotor integration than a classroom control condition among preschoolers. More broadly, in a meta-analysis of randomized controlled trials, Zhao et al. (30) reported that physical activity interventions were associated with marked improvements in children's visual perception, a core component of visuomotor integration. Another explanation involves the mediating role of fine motor coordination. Physical fitness may enhance fine motor coordination, which in turn supports visuomotor integration performance by enabling children to grip a pencil precisely and self-correct their movements to achieve accurate reproductions (10, 11, 31). In the present study, standing long jump exhibited the highest factor loading on the physical fitness construct at both time points, suggesting that it served as a strong representative indicator of this construct in the current sample. This finding is consistent with prior preschool research, in which horizontal jump similarly loaded strongly on the physical fitness construct (16). Rather than reflecting lower-limb power alone, jumping performance may capture broader neuromuscular control and coordinated motor output, which are developmentally relevant to visuomotor integration. In contrast, greater visuomotor integration does not necessarily translate into broader movement engagement (e.g., higher physical activity levels) or subsequent fitness gains. For instance, Flores et al. (32) found that visuomotor integration was not significantly associated with physical activity levels in preschoolers, even though visuomotor integration was correlated with gross motor skills.

This study has several limitations that warrant consideration in future research. First, generalizability may be limited because participants were recruited from a single kindergarten through convenience sampling. The findings should therefore be interpreted with caution and may not extend directly to preschool children from other regions, socioeconomic backgrounds, or educational settings. Future research employing larger, more representative samples across multiple regions could enhance external validity. Second, although age and sex were controlled in the analyses, several potentially important confounders were not assessed, including socioeconomic status, nutritional status (e.g., baseline BMI), habitual out-of-school physical activity, baseline cognitive functioning (e.g., IQ), parental support, and home cognitive stimulation. Because these factors may simultaneously influence the development of physical fitness and visuomotor integration, residual confounding cannot be fully ruled out. Future studies should measure and adjust for these variables to more clearly delineate the longitudinal association between physical fitness and visuomotor integration. Third, the two-wave cross-lagged panel design strengthened temporal inference but could not capture developmental trajectories or nonlinear change over time. In addition, strong autoregressive effects may attenuate cross-lagged estimates. Additionally, the present study did not examine other potential mediating variables that may underlie the association between physical fitness and visuomotor integration. Future research examining bidirectional associations between physical fitness and visuomotor integration through alternative mediating variables, such as visual perception and fine motor coordination, may further elucidate the underlying causal mechanisms.

## 5 Conclusions

The present study demonstrated that physical fitness was longitudinally associated with subsequent visuomotor integration in preschool children. These findings contribute to the growing body of longitudinal evidence on the link between physical fitness and visuomotor integration during early childhood.

## Data Availability

The raw data supporting the conclusions of this article will be made available by the authors, without undue reservation.
